# “These Are Just Stories, Mulder”: Exposure to Conspiracist Fiction Does Not Produce Narrative Persuasion

**DOI:** 10.3389/fpsyg.2018.00684

**Published:** 2018-05-23

**Authors:** Kenzo Nera, Myrto Pantazi, Olivier Klein

**Affiliations:** ^1^Center for Social and Cultural Psychology, Université Libre de Bruxelles, Brussels, Belgium; ^2^Department of Psychology, University of Cambridge, Cambridge, United Kingdom

**Keywords:** conspiracy theories, conspiracy fiction, conspiracy beliefs, conspiracy mentality, narrative persuasion, Extended Elaboration Likelihood Model

## Abstract

Narrative persuasion, i.e., the impact of narratives on beliefs, behaviors and attitudes, and the mechanisms underpinning endorsement of conspiracy theories have both drawn substantial attention from social scientists. Yet, to date, these two fields have evolved separately, and to our knowledge no study has empirically examined the impact of conspiracy narratives on real-world conspiracy beliefs. In a first study, we exposed a group of participants (*n* = 37) to an X-Files episode before asking them to fill in a questionnaire related to their narrative experience and conspiracy beliefs. A control group (*n* = 41) had to answer the conspiracy beliefs items before watching the episode. Based on past findings of both the aforementioned fields of research, we hypothesized that the experimental group would show greater endorsement of conspiracy beliefs, an effect expected to be mediated by identification to the episodes' characters. We furthermore hypothesized that identification would be associated with cognitive elaboration of the topics developed in the narrative. The first two hypotheses were disproved since no narrative persuasion effect was observed. In a second study, we sought to replicate these results in a larger sample (*n* = 166). No persuasive effect was found in the new data and a Bayesian meta-analysis of the two studies strongly supports the absence of a positive effect of exposure to narrative material on endorsement of conspiracy theories. In both studies, a significant relation between conspiracy mentality and enjoyment was observed. In the second study, this relation was fully mediated by two dimensions of perceived realism, i.e., plausibility and narrative consistency. We discuss our results, based on theoretical models of narrative persuasion and compare our studies with previous narrative persuasion studies. Implications of these results for future research are also discussed.

## Introduction

Conspiracy theories—explanations of (often socially significant) events involving the causal role of a deliberately malevolent group plotting in secrecy (Keeley, 1999)—are the focus of growing interest among social scientists. This increased interest stems from the fact that these theories are widespread in the contemporary world (e.g., Fenster, 1999; Taguieff, 2005; Aaronovitch, 2009; Bronner, 2013). Moreover, conspiracy theories can exert harmful outcomes, especially when they challenge the scientific consensus: anti-vaccination beliefs have become a serious problem for public health (Goertzel, 2010; Jolley and Douglas, 2014; Douglas et al., 2015), and the belief that anthropogenic global warming is a hoax has obvious deleterious implications for environment policies (Goertzel, 2010). While it is not clear if conspiracy theories are more popular today than they were before (cf. Van Prooijen and Douglas, 2017), authors argued that the development of the worldwide web since the late twentieth century has favored the spread and general visibility of conspiracy theories (e.g., Willman, 2002; Barkun, 2003; Kata, 2012; Bronner, 2013; Wood, 2013). These elements show the importance of understanding the mechanisms underpinning conspiracism.

What factors predict the endorsement of conspiracy theories (henceforth CTs)? One of the key findings in the social psychological research on the topic is that believing a CT is positively correlated with believing in other CTs (Goertzel, 1994). This finding lead to the hypothesis that CTs are part of a “monological belief system” (Goertzel, 1994), i.e., a belief system ignoring contradictory facts and ideas, in which CTs serve as mutually reinforcing evidence. Even though Wood et al. (2012) have shown that the correlation between conspiracy beliefs was observed even when they were contradictory, the idea that CTs are associated with a generic self-sustained belief system has been empirically supported by multiple studies (e.g., Wood et al., 2012; Van Prooijen et al., 2015; Franks et al., 2017). Along this line of research, measures of generic propensity to endorse CTs have been developed (e.g., Brotherton et al., 2013; Bruder et al., 2013; Imhoff and Bruder, 2014; Lantian et al., 2016). Other lines of research have focused on personality-related variables associated with the endorsement of CTs (e.g., Goertzel, 1994; Darwin et al., 2011; Douglas and Sutton, 2011; Newheiser et al., 2011; Marchlewska et al., 2017), as well as on generic cognitive biases associated with conspiracy mentality and CT endorsement (e.g., van Prooijen and van Dijk, 2014; Brotherton and French, 2015; Douglas et al., 2016). Finally, political and contextual factors involved in the endorsement of CTs have also been investigated, although they have attracted less attention (van Prooijen and Jostmann, 2013; Mashuri and Zaduqisti, 2015; van Prooijen and van Dijk, 2014; Van Prooijen et al., 2015; Uenal, 2016; Van Prooijen and Douglas, 2017).

A question that has not been explored yet is the impact that culture, and especially fiction, exerts on the development of conspiracy beliefs. Since conspiracies of all kind are a recurring theme in fiction, this issue undoubtedly deserves more attention. North American culture—probably the most widespread worldwide—has been fascinated by conspiracies for decades (Fenster, 1999; Coale, 2003), and countless best-selling fictions—novels, movies, series, video games—are based on conspiratorial themes^1^. Thus, the idea that some (often dreadful) reality is concealed from the public eye by malevolent forces seems very salient in fiction (Fenster, 1999; Coale, 2003; Uscinski and Parent, 2014).

Given the prevalence of conspiratorial themes in fiction, a reasonable hypothesis is that conspiracy-inspired fictions could affect the endorsement of conspiracy beliefs in real life. Prior research has shown that explicitly fictional narratives can influence people's beliefs about the real world (e.g., Green and Brock, 2000), even if the information contained in these narratives is explicitly incorrect (Marsh et al., 2003; Marsh and Fazio, 2006; Eslick et al., 2011). Other studies demonstrated that mere exposure to CTs tends to increase their endorsement (Douglas and Sutton, 2008; Jolley and Douglas, 2014; van der Linden, 2015). Such works highlight the strength and persistence of misinformation, and are consistent with Gilbert's (1991) theory according to which people are prone to believe any information they encounter (see also, Kissine and Klein, 2014; Pantazi et al., 2018). To our knowledge, however, the impact of conspiracy fiction on conspiracy beliefs has not been investigated. In the current study, we experimentally explored this question, based on existing theoretical models about narrative persuasion.

Narrative persuasion can be briefly defined as the impact of narratives on the attitudes, beliefs and behaviors of individuals exposed to them (e.g., Green and Brock, 2000; Moyer-Gusé and Dale, 2017). Narratives, on the other hand, can be defined as “any cohesive and coherent story with an identifiable beginning, middle, and end that provides information about scene, characters, and conflict, raises unanswered questions or unresolved conflict, and provides solution” (Hinyard and Kreuter, 2007, p. 778). The fundamental assumption of narrative persuasion theories is that fictional narratives, even when primarily designed to entertain, can have a persuasive impact on individuals, for stories often implicitly include arguments on the social topics or actors they portray (Green and Brock, 2005). Narrative persuasion is anything but a frivolous, anecdotal research field, for at least two reasons. First, as Green and Brock (2000) put it, “public narrative predominates over public advocacy: novels, films, soap operas, […] command far more waking attention than do advertisements, sermons, editorials […]” (p. 701). Second, narratives are a powerful mean of persuasion (e.g., Fisher, 1984; Moyer-Gusé and Dale, 2017). Indeed, the phenomenon of narrative persuasion has been established in numerous empirical studies (e.g., Schofield and Pavelchak, 1989; Gerrig and Prentice, 1991; Butler et al., 1995; Prentice et al., 1997; Strange and Leung, 1999; Green and Brock, 2000; Marsh and Fazio, 2006; Fazio and Marsh, 2008).

The three prominent models of narrative persuasion are the *Transportation Imagery Model* (henceforth TIM; Green and Brock, 2000), the *Extended Elaboration Likelihood Model* (henceforth E-ELM; Slater and Rouner, 2002), and the narrative engagement model (Busselle and Bilandzic, 2008). According to Green and Brock's TIM, narrative persuasion results from a mechanism called psychological transportation: the more a person feels emotionally and cognitively transported into the narrative, the more he or she is likely to develop story-consistent beliefs and attitudes.

The E-ELM, while acknowledging the role of *absorption*—a concept very close to that of *transportation*—also attributes significant importance to identification with narrative characters in the persuasion process. Absorption and identification with characters are thought to facilitate the narrative persuasion process by impeding the development of a critical thinking while a person experiences the narrative (Slater and Rouner, 2002; Dal Cin et al., 2004). Moreover, the experience of identification provides an enjoyment that lowers the motivation necessary to elaborate critical thoughts (Cohen, 2001; Iguarta, 2010). For some researches, character identification is actually a key mediator of the narrative persuasion process (Moyer-Gusé, 2008; Iguarta, 2010; Moyer-Gusé and Nabi, 2010; de Graaf et al., 2012; Iguarta and Barrios, 2012; Iguarta and Casanova, 2016). Note that such mediation is also congruent with social cognitive theory (Bandura, 2002), according to which a stronger identification with a role model leads to improved observational learning. Furthermore, identification is associated with greater cognitive elaboration (Iguarta, 2010) i.e., the process of thinking about the topics developed in the narrative or the “intensity of information processing” (Suckfüll and Scharkow, 2009, p. 274). On the other hand, identification is thought to decrease counterarguing i.e., the emission of negative comments about the persuasive message (Cohen, 2001; Dal Cin et al., 2004; Iguarta, 2010; Moyer-Gusé and Nabi, 2010).

Lastly, the narrative engagement model (Busselle and Bilandzic, 2008) focuses on how different dimensions of perceived realism affect the transportation process. Specifically, this approach posits that while fictionality (i.e., the fact that the story is fictitious vs. based on real events) does not necessarily affect transportation, narrative realism (i.e., internal consistency of the story) and external realism (i.e., how the narrative appears to reflect potential real-life situations) can disrupt, or foster, the transportation process.

Although the above-mentioned models are general models of narrative persuasion, most studies have focused on the effects of narratives on normative attitudes, beliefs and behaviors, e.g., in the field of health communication (e.g., Slater and Rouner, 2002; Dal Cin et al., 2004; Moyer-Gusé, 2008; Murphy et al., 2013; Iguarta and Casanova, 2016). Very few studies have relied on a narrative persuasion model to examine the impact of narratives conveying controversial messages (Slater et al., 2006; Iguarta and Barrios, 2012). CTs are, by definition, controversial. Even though they are not *per se* qualified as false—there have been, indeed, conspiracies in history—they are more often than not considered as intrinsically flawed explanations, regardless of their hypothetical veracity (Keeley, 1999) and studies have shown that people who endorse them are perceived rather negatively (Klein et al., 2015; Lantian et al., 2018). Hence, the very few studies exploring the impact of narratives conveying controversial attitudes are, in the context of our work, particularly interesting, for they empirically suggest that the narrative persuasion theoretical models might be generalizable to any type of persuasive content, including conspiracy beliefs.

In one study, Slater et al. (2006) examined if a narrative experience could affect support for controversial political attitudes such as support for the death penalty and gay marriage. Specifically, they exposed half of their participants to an episode of the *Law and Order* television show conveying support for the death penalty, while the other half was exposed to a shortened version of *If These Walls Could Talk II*, conveying support for gay marriage. In accordance with their hypotheses, exposition to the *Law and Order* episode increased participants' support for the death penalty, and suppressed the relation between policy support and prior ideology (measured on the conservative/liberal continuum). However, such persuasion was not observed for the narrative conveying support for gay marriage. According to the authors, at the time of the study, the issue of gay marriage was very salient in the US, making the persuasion process harder to achieve. Iguarta and Barrios (2012) report more consistent findings concerning the effects of narratives with controversial message. In their experiment they found that participants who had just watched *Camino* (Fesser, 2008)—a controversial movie that criticizes religion and especially the *Opus Dei* organization—reported more negative attitudes toward religion and the organization than the control group, who reported attitudes before watching the movie. Moreover, watching the movie suppressed the relation between attitudes and prior political ideology, which was observed in the control group. Besides, the persuasion effect was mediated by the strength of participants' identification with the main protagonist of the movie.

## Study 1

### Hypotheses

In view of the literature reviewed above, our first hypothesis is that exposure to a fictional narrative that presents various conspiracies will have an impact on the viewer's conspiracy beliefs. This stems directly from past narrative persuasion studies (e.g., Green and Brock, 2000; Slater et al., 2006; Appel and Richter, 2010; Iguarta, 2010; Iguarta and Barrios, 2012).

*H*_1_: Participants exposed to an X-Files episode conveying a conspiracist worldview will endorse related conspiracy beliefs more than participants who do not view the episode.

Our second hypothesis is that character identification will mediate the persuasive impact of a conspiracy-based narrative. Identification was included as a mediating variable in our experiment, since character identification has been previously found to mediate the impact of a controversial movie on people's attitudes. Following Iguarta and Barrios (2012), we tested this mediation hypothesis through a correlation coefficients comparison.

*H*_2_: There will be a significantly stronger correlation between character identification and conspiracy beliefs in the experimental than in the control group.

Our third and last hypothesis is correlational, and predicts a positive link between character identification and cognitive elaboration—how much the subject thought about the topic developed in the narrative while experiencing it (Petty and Cacioppo, 1986). This effect has been speculated by Cohen (2001) and empirically demonstrated by Iguarta (2010). In the context of our study, we examined whether identifying with a protagonist of a conspiratorial narrative could stimulate reflection about the topics developed in the narrative, i.e., conspiracies.

*H*_3_: A stronger character identification will be associated with a stronger cognitive elaboration.

### Materials and methods

#### Participants

Our study was completed by 81 Belgian participants, three of which were eliminated from the dataset for not answering correctly the control questions (36 women, 45 men; *M*_*age*_ = 25.8 years old, range 18–55 years old, *SD* = 6.7 years). The sample was 54.7% students (*n* = 41), 32% employees, 10.7% unemployed *(n* = 8), 2.7% self-employed (*n* = 2). The mean for political orientation was 3.19 (*SD* = 1.13) on a scale going from 1 (far left) to 7 (far right).

In this experiment, sample size was not calculated before data collection. We carried out a *post hoc* power analysis using the G^*^power software (Faul et al., 2007) that revealed that the sample size provided a power of 0.60 to detect a small effect (*d* = 0.50) with two tailed tests.

#### Procedure

Our study design is similar to the one used by Iguarta and Barrios (2012), which was itself inspired by earlier similar works (e.g., Butler et al., 1995; Koopman et al., 2006; Iguarta, 2010, Study 3). The main differences are that our study was conducted online, and examined the impact of a 44 min long TV show episode, while the aforementioned studies examined the impact of feature films. The selected episode was particularly eclectic in its conspiratorial inspirations, involving evil elites plotting world domination, concealed alien contacts, secret military investigation, assassinations of “inconvenient witnesses,” generalized government surveillance, and controlled mass media.

The video and questionnaire were uploaded on the LimeSurvey platform (http://www.LimeSurvey.org). The recruitment of participants was made online, via a Facebook page regularly used to share experiment advertisements. After an introductory message informing participants that the experiment tested the enjoyment of mainstream series, participants were asked to indicate the last digit of their phone number. This was to allocate participants to the two conditions randomly. In the experimental condition, participants answered items measuring endorsement of conspiracy beliefs and conspiracy mentality after viewing the episode. In the control condition, those questions were asked before the viewing. The episode was included in the questionnaire as a link to a private streaming platform, with a message stating that the questionnaire should be carried on only after viewing the whole episode. Participants were encouraged not to pay attention to anything in particular, and watch the TV show episode as they would in their leisure time. After viewing the episode, participants in both conditions had to answer two control questions and items related to their experience of the narrative: enjoyment, cognitive elaboration, attentional focus, and character identification. In the experimental condition, the items related to conspiracy beliefs were answered after the aforementioned measures. The questionnaire ended with measures of socio-demographic variables. Before completing the questionnaire, participants could write a comment about the experiment, and were given the first author's e-mail in case they wanted to know more about the research.

#### Variables and measures

In most narrative persuasion studies, beliefs and attitudes are assessed with a single item (e.g., “Religion is an obstacle to living a full life,” Iguarta and Barrios, 2012; “Malls aren't safe places,” Green and Brock, 2000). We used similar single items related to specific conspiracy beliefs developed in the episode. Several other variables were included in order to test our aforementioned hypotheses. Note that all continuous variables were measured using seven point Likert scale.

##### Story related conspiracy beliefs

Three items were created to assess endorsement of conspiracy beliefs that were particularly salient in the episode:

1. “Laws limiting privacy and individual freedom in the name of state security are in fact tools of the elite to enslave the population.”
2. “Some groups are in possession of extremely advanced military technology, whose existence is kept secret.”
3. “To protect some secrets, Western governments are willing to perform illegal actions, including assassinations.”

The three items were also averaged into a single variable that had acceptable internal reliability (*α* = 0.69). Conspiracy beliefs served as dependent variables in H1 and H2.

In addition, we included the conspiracy mentality scale (Imhoff and Bruder, 2014). This twelve-items scale has been thought to represent a stable political attitude. Nevertheless since it has never, to our knowledge, been subject to such an experimental manipulation we included it in our questionnaire as a dependent variable to test H1 and H2. In case the experimental manipulation has no effect on conspiracy mentality scores, the variable could be considered as potential moderator in the analyses.

##### Conspiracy mentality scale (α = 0.86)

The twelve items of this scale (Imhoff and Bruder, 2014) measure general beliefs that underpin conspiracy mentality (e.g., “There are many very important things happening in the world about which the public is not informed”). For the purpose of the study, the English version of the questionnaire was translated into French^2^.

##### Identification scale (α = 0.91)

The thirteen items measured merging with the character (6 items), cognitive (4 items), and emotional empathy (3 items). We translated the English version of the scale (Iguarta, 2010) into French. Since there are few main protagonists in the selected episode (mostly the Mulder/Scully team) we asked participants to mention whom they identified with before filling the scale (“The episode involves different characters, which one did you identify to the most?”). A principal components analysis with varimax rotation performed on our sample yielded three dimensions of identification (for statistics, see Appendix 1), congruent with Iguarta and Barrios' study 2012: cognitive empathy (3 items), emotional empathy (4 items), merging with the character (6 items). Identification was the independent variable in H2 and H3.

##### Cognitive elaboration (α = 0.81)

Cognitive elaboration is defined by Petty and Cacioppo (1986) as the intensity of the reflection a subject has about the content of a narrative (e.g., “I reflected about the topics dealt with in the narrative”). The four items were created in Spanish by Igartua and Paèz (1997) before being translated into English by Iguarta (2010), and were translated into French for the experiment. Cognitive elaboration was the dependent variable in H3.

##### Enjoyment of the episode

It was measured via a single item, “How much did you enjoy the episode you just watched?”. This measure was included for exploratory analyses.

##### Attentional focus (α = 0.92)

We designed four items measuring how focused participants were during the viewing (e.g., “My thought were only focused on the episode”). This measure was also included in the questionnaire for exploratory analyses.

##### Sociodemographic variables

Gender (M/F), age, economic situation, political position (1 = far left, 7 = far right).

##### Control questions

The two control questions were the following: “Have you ever seen this episode?” (Yes/No) and “What happens to Sveta, the young girl abducted by the aliens, at the end of the episode?” (multiple choice).

##### The exposition material: X-Files episode “my struggle” (Carter, 2016)

The narrative material to which participants were exposed was the first episode of the tenth season of the X-Files, in its original version with French subtitles. The choice of the narrative was not easy, as there are countless popular fictions based on conspiracies. We chose our material based on three criteria:

1)The material should not just involve a conspiracy, but should somehow develop a “conspiracist worldview” (e.g., with insistence on the collusion of mass media, mentions of evil elites, secrecy and government malevolence, see Koltko-Rivera, 2004; Franks et al., 2017). Such features would make the narrative material congruent with the fact that CTs are mostly part of a general belief system.

2)The material should be mainstream, enjoyable by most people. We therefore disqualified anything too graphic, “artsy” or complex.

3)The material should not be too long for practical reasons (i.e., to avoid attrition), but should be long enough to provide a significant narrative experience. This taken in account, a 45 min episode seemed an adequate exposition material.

Considering those three criteria, popular TV show *The X-Files*, whose motto—“the truth is out there”—reflects its various conspiracy oriented scenarios, appeared as an ideal candidate. Moreover, “the X-Files” was very popular worldwide during its years of airing, and won several awards in recognition for its qualities. The chosen episode is a mix of various evil schemes articulated around a vast, global conspiracy. In this episode, the two protagonists—Fox Mulder and Dana Scully—discover the existence of a global conspiracy plotted by a worldwide elite using advanced alien technology to take over the USA. In the investigation, they are assisted by “independent journalist” Tad O'Malley, host of the fictional web show “The Truth Squad”. The episode lasts for 44 min, which is a remarkably long exposition material for a narrative persuasion study—most of them focusing on short clips (e.g., educational or advertisement clips), with the notable exceptions of studies examining the impact of movies (e.g., Iguarta, 2010; Iguarta and Barrios, 2012).

### Results

SPSS data file and material can be found on the following address: https://osf.io/24zf5/.

**Figure 1:**
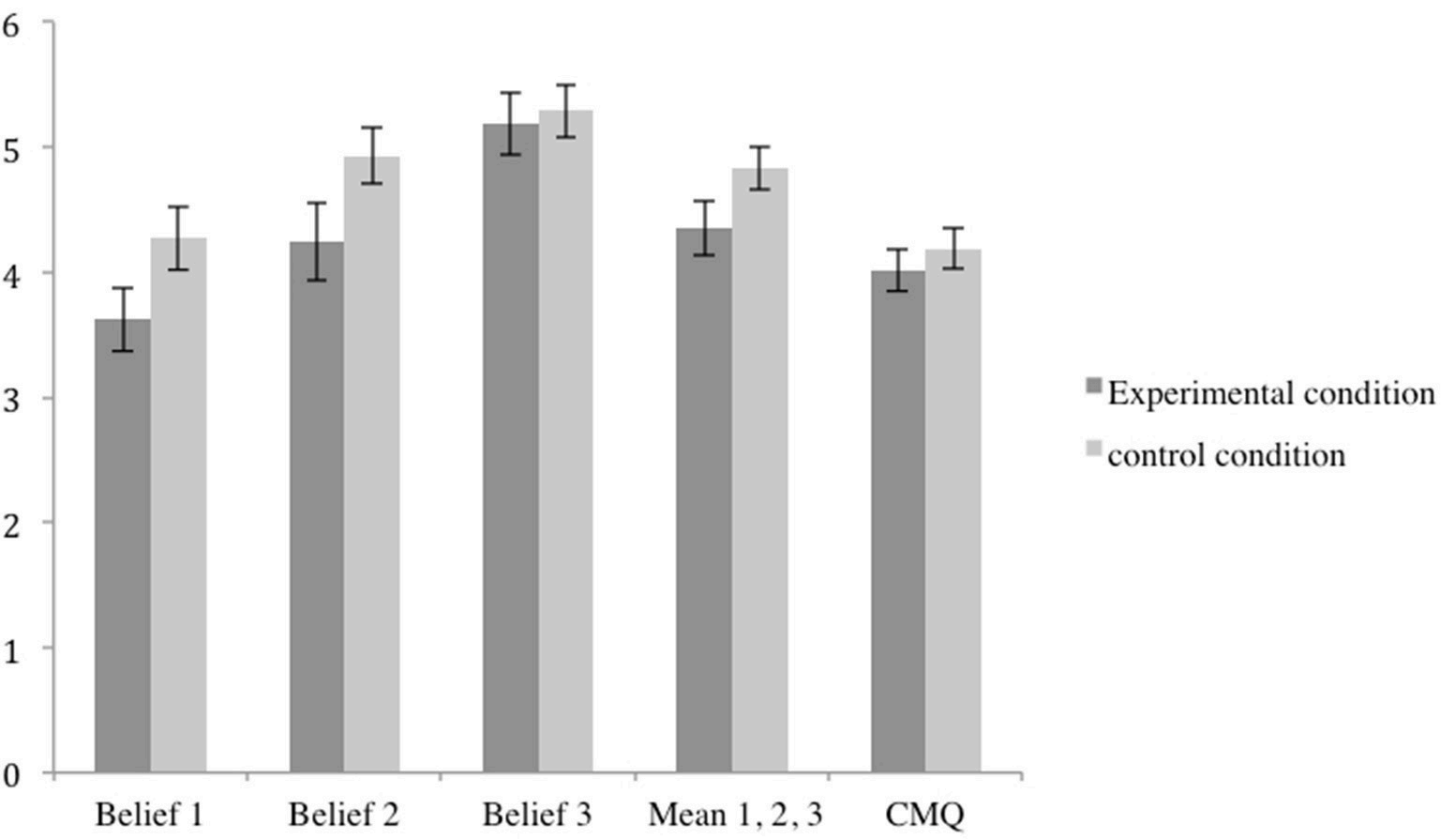
Conspiracy beliefs and mentality by condition with confidence intervals (*α* = 5%). Belief 1: “Laws limiting privacy and individual freedom in the name of state security are in fact tools of the elite to enslave the population.” Belief 2: “Some groups are in possession of extremely advanced military technology, whose existence is kept secret.” Belief 3: “To protect some secrets, occidental governments are willing to perform illegal actions, including assassinations.” CMQ, Conspiracy Mentality Questionnaire (Imhoff and Bruder, 2014).

Our first hypothesis was that participants in the experimental condition would show stronger agreement with conspiracy beliefs than participants in the control condition. Separate one-way ANOVAs were performed for the different conspiracy beliefs variables. These analyses revealed that, surprisingly, the control group showed slightly greater agreement with two conspiracy beliefs associated with the episode than did the experimental group (see Figure 1). This difference approached significance for two of the three variables [“Laws limiting privacy and individual freedom in the name of state security are in fact tools of the elite to enslave the population”, *F*_(1, 76)_ = 3.27; *p* = 0.074, *M*_*control*_ = 4.27, *SE* = 0.25; *M*_*experimental*_ = 3.62, *SE* = 0.25, η^2^_*partial*_ = 0.04; and “Some groups are in possession of extremely advanced military technology, whose existence is kept secret.”, *F*_(1, 76)_ = 3.39, *p* = 0.069, *M*_*control*_ = 4.93, *SE* = 0.22; *M*_*experimental*_ = 4.24, *S.E* = 0.31, $\eta_{partial}^{2}$ = 0.04]. This “boomerang effect” (see Brehm and Brehm, 1981) is the exact opposite of what we expected. A similar marginally significant difference between groups was also detected when averaging the three individual beliefs into one variable, *F*_(1, 76)_ = 3.02, *p* = 0.086, *M*_*control*_ = 4.83, *SE* = 0.17; *M*_*experimental*_ = 4.35, *SE* = 0.22, $\eta_{partial}^{2}$ = 0.04. Conspiracy mentality scores did not differ between conditions, *F*_(1, 76)_ = 0.53, *p* = 0.47, and neither did the belief “To protect some secrets, occidental Western governments are willing to perform illegal actions, including assassinations.”, *F*_(1, 76)_ = 0.1, *p* = 0.75.

To examine whether these unexpected results could be due to a failure in random allocation, we performed separate one-way ANOVAs (and a Chi square test for gender) to check for possible differences between conditions on all measured variables. No differences were found for identification, *F*_(1, 76)_ = 0.068, *p* = 0.79, cognitive elaboration, *F*_(1, 76)_ = 0.51, *p* = 0.48, attentional focus, *F*_(1, 76)_ = 0.16, *p* = 0.69, enjoyment, *F*_(1, 76)_ = 0.70, *p* = 0.41, gender, $\chi_{\left(1\right)}^{2}$ = 0.62, *p* = 0.66, age, *F*_(1, 76)_ = 0.29, *p* = 0.59, or political orientation, *F*_(1, 76)_ = 0.39 *p* = 0.54. Hence, it seems unlikely that failure of random sampling could have caused the observed results.

These unexpected results concerning our first hypothesis made our mediation hypothesis seem moot, for it sounded illogical that a strong identification with a character could mediate the rejection of beliefs held by the character. Nevertheless, we performed Fisher's r-to-z test to compare the correlation between identification and narrative-related conspiracy beliefs in the experimental and the control group. Unsurprisingly, the test result was not significant, *Z* = 0.16, *p* = 0.44, *ns*.

Our third and last hypothesis was that stronger identification would lead to more cognitive elaboration. A linear regression using identification as independent variable and cognitive elaboration as dependent variable confirmed this hypothesis, *β* = 0.44, *t*_(76)_ = 4.24*, p* < 0.001. Noticing that cognitive elaboration was strongly correlated to conspiracy mentality, *r* = 0.34, *p* = 0.002, we examined the possibility of an interaction between identification and conspiracy mentality. Both independent variables were centered and multiplied into an interaction variable. However, no interaction was found, *β* = −0.05, *t*_(74)_ = −0.49, *p* = 0.62.

#### Exploratory analyses

Since the matrix of correlations between all measured variables (see Table 1) showed significant relations between conspiracy mentality and variables related to the narrative, we performed several exploratory analyses on our data.

**Table 1:** Correlations between all measured variables (study 1).

	**1**	**2**	**3**	**4**	**5**	**6**	**7**	**8**	**9**
1.Enjoyment	1	0.24^*^	0.11	0.2	0.28^*^	0.12	0.14	0.27^*^	0.13
2.Conspiracy mentality scale	0.24^*^	1	0.76^*^	0.15	0.06	0.08	0.21	0.34^**^	0.34^**^
3.Narrative related CTs	0.11	0.76^**^	1	0.23^*^	0.16	0.14	0.24^*^	0.35^**^	0.23^*^
4.Identification	0.20	0.15	0.23^*^	1	0.8^**^	0.76^**^	0.88^**^	0.2	0.44^**^
5.Emotional empathy	0.28^*^	0.06	0.16	0.80^**^	1	0.51^**^	0.56^**^	0.19	0.29^**^
6.Cognitive empathy	0.12	0.08	0.14	0.76^**^	0.51^**^	1	0.48^**^	0.16	0.47^**^
7.Merging	0.14	0.21	0.24	0.88^**^	0.56^**^	0.48^**^	1	0.16	0.33^**^
8.Attentional focus	0.27^*^	0.34^**^	0.35^**^	0.2	0.19	0.16	0.16	1	0.39^**^
9.Cognitive elaboration	0.13	0.34^**^	0.23^*^	0.44^**^	0.29^**^	0.47^**^	0.33^**^	0.39^**^	1

* p < 0.05

** *p < 0.001*.

Conspiracy mentality was a significant predictor of enjoyment, *β* = 0.24, *t*_(76)_ = 2.22, *p* = 0.03, attentional focus, *β* = 0.34, *t*_(76)_ = 3.14, *p* = 0.002, and cognitive elaboration, *β* = 0.34, *t*_(76)_ = 3.17, *p* = 0.002. Since conspiracy mentality seems to be a stable socio-political attitude, this relationship is probably explained by the fact that conspiracy mentality favored enjoyment of the narrative, attentional focus, and thinking of issues dealt with in the narrative. Note, however, that conspiracy mentality did not predict the strength of character identification, *t*_(76)_ = 0.85, *p* = 0.40, although it was a marginally significant predictor of the merging dimension of identification, *β* = 0.21, *t*_(76)_ = 1.83, *p* = 0.07.

Scully being the skeptic in the duo, and Mulder the “conspiracy believer,” we examined if people tended to identify with the character who is seemingly more compatible with their own belief system. A logistic regression showed that among the participants who identified with Dana Scully or Fox Mulder (*n* = 65), those with higher conspiracy mentality scores were more likely to identify with Mulder, *β* = 0.97, *SE* = 0.37, χ^2^_(1)_ = 7.05, *p* = 0.008. Furthermore, participants identifying with Mulder had significantly higher conspiracy mentality scores than participants identifying with Scully, *F*_(1, 63)_ = 6.87, *p* = 0.01, *M*_*Scully*_ = 3.86, *SE* = 0.15; *M*_*Mulder*_ = 4.53, *SE* = 0.21. Unsurprisingly, gender was also a significant predictor of character identification, participants being more likely to identify with the character of their gender *β* = 1.14, *SE* = 0.44, χ^2^_(1)_ = 7.91, *p* = 0.005. We performed a second logistic regression including an interaction term, however no interaction was observed between gender and conspiracy mentality, *β* = –0.37, *SE* = 0.37, χ^2^_(1)_ = 0.10, *p* = 0.32, *ns*. These data indeed corroborate the hypothesis that similarity between subject and character facilitates identification (e.g., Maccoby and Wilson, 1957; Cohen, 2001; Eyal and Rubin, 2003).

### Discussion

Our first experiment yielded unexpected results, contradicting our main hypotheses. Indeed, exposure to an episode of the X-Files seemed to decrease, rather than increase, conspiracist beliefs. These result may indicate the existence of what in the attitude-change literature has been called a “boomerang effect” (Hovland et al., 1953), a tendency for people to react to persuasive messages by endorsing attitudes that are opposite to those advocated in such messages. A dominant explanation of the boomerang effect is psychological reactance (Brehm and Brehm, 1981): Thus, people may perceive the persuasive message as an attempt to restrict their freedom of thought or expression and may therefore reassert this freedom by rejecting the attitude advocated in the message. An alternative, and possibly complementary mechanism is counterarguing, which can be defined as the cognitive process of emitting negative comments about the content of the narrative (Slater et al., 2006). It is plausible that participants had critical thoughts about the beliefs held by the characters, despite the fact that they enjoyed the episode (see Table 2 for descriptive statistics about the narrative experience). Nonetheless, note that one of the strength of narratives as persuasive devices, is that they may minimize counterarguing due to the audience being transported in the narrative and not necessarily realizing the persuasive nature of the message (Slater et al., 2006; Moyer-Gusé et al., 2011; Niederdeppe et al., 2012). However, as this finding was unpredicted and did not reach statistical significance, it may be spurious and should be further corroborated.

**Table 2:** Descriptive statistics for all measured variables (study 1).

**Variable name**	**Mean**	***SD***
Enjoyment	4.78	1.34
Conspiracy mentality scale	4.11	1.03
Narrative related CTs	4.6	1.23
Identification	3.55	1.23
Emotional empathy	3.77	1.58
Cognitive empathy	4.44	1.59
Merging	2.89	1.37
Participant's focus	4.65	1.38
Cognitive elaboration	4.9	1.28

The exploratory analyses showed an unexpected, albeit interesting, relation between conspiracy mentality and enjoyment with the narrative. This suggests that, while not necessarily increasing conspiracy beliefs, this kind of narratives may be a source of enjoyment for people who already espouse such beliefs. This in turn may encourage selective exposure to such narratives. Moreover, participants scoring high on the conspiracy mentality scale tended to pay more attention while watching, and reflect more about the topics dealt in the narrative. These results suggest that the prior belief system of the recipients affects the processing of narratives, which is a causal direction that has been scarcely investigated in narrative persuasion research. This may be related to the finding that prior knowledge of the topics dealt in a narrative favors narrative transportation (Green, 2004).

A major limitation of this experiment is that given the observed effect sizes, its sample size provides low statistical power, which may explain the absence of significant results. In order to establish the reliability of these findings a replication was carried out on a larger sample.

## Study 2

Preregistration of the study, as well as the SPSS data file, can be found on the Open Science Framework, at the following address: https://osf.io/24zf5/.

### Hypotheses

Based on the results of Study 1, we hypothesized that given a larger sample, we would observe a significant boomerang effect. Therefore, we included in the questionnaire measures of two potential mediators: counterarguing, and psychological reactance to the experiment setting.

*H*_1_: Participants exposed to an X-Files episode conveying a conspiracist worldview will endorse related conspiracy beliefs *less* than participants who do not view the episode.

*H*_2_: The effect predicted in *H*_*1*_ will be mediated by either counterarguing or psychological reactance to the experimental setting, or both.

In addition, we explored the positive relation between conspiracy mentality and enjoyment of the narrative, and included as potential mediators three dimensions of perceived realism (Hall, 2003), i.e., perceived plausibility, perceived factuality, and perceived narrative consistency. These constructs have been shown to be associated with emotional involvement in the narrative (Cho et al., 2012). Since higher score of conspiracy mentality suggests greater congruence between participants' belief system and worldview conveyed in the narrative, we hypothesized that perceived realism would mediate the relation between conspiracy mentality and enjoyment of the narrative.

*H*_3_: Participants scoring high on the conspiracy mentality questionnaire will tend to enjoy the narrative more.

*H*_4_: The relation between conspiracy mentality and enjoyment will be mediated by the perceived realism of the narrative.

### Materials and methods

#### Participants

216 participants from the UK recruited via Prolific Academic participated in the experiment, among which 50 were excluded for not answering correctly to at least one of the control checks (100 women; *M*_*age*_ = 36.9 years old, range 18–73 years old, *SD* = 11.97). Participants covered a range of educational backgrounds: secondary school (10.8%, *n* = 18), College/A-Levels (30.1%, *n* = 50), undergraduate degree (40.4%, *n* = 67), graduate degree (13.9%, *n* = 23), and doctorate degree (3%, *n* = 5), while 1.2% of subjects had no formal qualification (*n* = 2). The mean for political orientation was 3.5 (*SD* = 1.17) on a scale going from 1 (= far left) to 7 (far right).

Required sample size was calculated using G^*^Power prior to data collection. In order to achieve a statistical power of 0.80 to detect an effect size of *d* = 0.4 (which corresponds to the effect sizes observed in the first experiment, and is also the mean effect size in social psychology, see Richard et al., 2003) with one-tailed tests, 156 subjects were needed. The actual sample size provided a power of 0.82. In quantitative psychology, a statistical power of 0.80 is usually considered to be an acceptable balance between minimal beta risk and the investigator's limited resources (Cohen, 1988).

#### Procedure

The procedure was the same as for study 1, except that the online questionnaire was designed in English, and modified in accordance with the new hypotheses. Moreover, in order to gather a larger sample of participants, some items were removed. Furthermore, the exposure material was considerably shorter, and consisted in the last 8 minutes of the X-Files episode. Before watching, participants were asked to carefully read a recap of the episode. In the experimental condition, participants responded to the items measuring endorsement of conspiracy beliefs related to the narrative after viewing of the episode, while in the control condition, participants responded to these items before the viewing. After watching the video, participants filled out a questionnaire including the following measures: control checks, enjoyment, attentional focus, counterarguing, dimensions of perceived realism (plausibility, factuality, narrative consistency), endorsement of CTs related to the video (in the experimental condition) psychological reactance to the experimental setting, conspiracy mentality, generic interest in CTs, and political affiliation. The control checks consisted in two questions about the episode (“Have you ever seen this episode?” and “What happens to Sveta, the young woman who was abducted several times, at the end of the video?”). Two attention checks were included in the questionnaire (“please check box 6”). After completing the questionnaire, participants could write an open comment and were given the main author's e-mail address for further information on the experiment. The completion of the experiment took approximately 14 min, and participants who correctly answered to the control and attention checks were paid £1.25.

#### Variables and measures

##### CTs related to the episode (α = 0.76)

The two items that yielded the results we aimed to replicate were kept and translated in English. Given previous results, we replaced the item “Laws limiting privacy and individual freedom in the name of state security are in fact tools of the elite to enslave the population” with “Above governments, there are organizations that secretly organize worldwide chaos (international conflicts, financial crises,…) for their own profit,” which is one of the main points of the characters' theory in the video.

##### Attentional focus (α = 0.75)

The same scale as above was used, but the item “My thoughts were entirely directed at the episode” was removed to shorten the questionnaire.

##### Counterarguing (α = 0.90)

Existing measures of counterarguing were conceived for explicitly persuasive message (e.g., Moyer-Gusé and Nabi, 2010). We therefore created new items adapted to our material, based on the theoretical characteristics of counterarguing (Slater et al., 2006). Four items were designed (e.g., “While I was watching the video, I thought that the theory advocated by the characters was ludicrous”).

##### Perceived plausibility (α = 0.88)

Plausibility can be defined as the degree to which individuals' consider that a narrative could *possibly* happen in real life (Hall, 2003). Before measuring perceived plausibility of the narrative, subjects were asked to assess the extent to which they thought that different elements of the story corresponded to a potential real life situation (“US government chasing and assassinating people who are aware of state secrets,” “US Government concealing and using extraterrestrial technology,” and “Secret international organization planning to take over the US, and the world”). The purpose of these measures was to avoid a floor effect on responses to the four items adapted from Cho et al. (2012) measuring perceived overall plausibility (e.g., “overall, the video shows things that could happen in real life”), and hence eschew plain rejection on the basis of some elements of the story (e.g., references to alien technology). Items measuring overall plausibility were preceded by the sentence: “Considering your answers to the previous question, please respond to the statements below by checking the value that corresponds to your opinion”.

##### Perceived factuality (α = 0.88)

Factuality refers to the degree to which a narrative is perceived to be based on facts (Hall, 2003). Just like plausibility, measure of overall factuality was preceded by three items that assessed the perceived factuality of the three previously stated elements of the plot. Three items were adapted from Cho et al. (2012) to measure perceived overall factuality (e.g., “Many elements in the video are based on facts”). Items were introduced by the same sentence as the one preceding items measuring overall plausibility.

##### Narrative consistency (α = 0.78)

Narrative consistency designates the degree to which a narrative is perceived to be coherent, without internal contradictions (Hall, 2003). Four items derived from Cho et al. (2012) were designed (e.g., “The story portrayed in the video made sense”).

##### Psychological reactance to the experimental setting (α = 0.83)

Since existing psychological reactance measures are aiming at the persuasive content of an exposure material (e.g., Lindsey, 2005; Dillard and Shen, 2005), three items were designed to measure the sense of being manipulated by the experimental setting (e.g., “As I filled out the questionnaire, I was annoyed by the feeling that the experimenter was trying to influence my opinions”), based on the main characteristic of psychological reactance, which is a disagreeable feeling of being pressured to adopt specific attitudes, beliefs or behavior (Quick, 2012).

##### Conspiracy mentality questionnaire (α = 0.87)

The CMQ (Bruder et al., 2013) is a five-item measure of generic propensity to endorse CTs (e.g., “Government agencies closely monitor all citizens”), highly correlated to the 12 items conspiracy mentality scale (Imhoff and Bruder, 2014) used in study 1. We used it here for the sake of brevity.

##### Sociodemographic variables

Gender (M/F), highest education level, age, political position (1 = far left, 7 = far right).

### Results

The correlation matrix and descriptives statistics for all measured variables are reported in Tables 3, 4. To examine if exposure to the X-Files video was associated with lesser endorsement of CTs related to the narrative, we performed separate one-way ANOVAs to examine the impact of the viewing on the endorsement of the three conspiracy beliefs related to the story, and the averaged score of the three items. All tests were performed while controlling the effect of conspiracy mentality. No differences between experimental and control groups were found, for any of the dependent variables (see Table 5). Therefore, neither the “boomerang” nor the “persuasive effect” was supported by our analyses.

**Table 3:** Correlations between all measured variables (study 2).

	**1**	**2**	**3**	**4**	**5**	**6**	**7**	**8**	**9**
1.CMQ	1	0.69^**^	0.27^*^	0.1	0.57^**^	0.55^**^	0.30^**^	−0.38	−0.01
2.Narrative related CTs	0.69^**^	1	0.27^**^	0.15	0.55^**^	0.48^**^	0.29^**^	−0.43	−0.12
3.Enjoyment	0.27^*^	0.27^**^	1	0.38^**^	0.51^**^	0.39^**^	0.5^**^	−0.55	−0.08
4.Attentional focus	0.1	0.15	0.38	1	0.2	0.11	0.34	−0.08	−0.15
5.Plausibility	0.57^**^	0.55^**^	0.51^**^	0.20^*^	1	0.59^**^	0.38^**^	−0.67	−0.1
6.Factuality	0.55^**^	0.48^**^	0.39^**^	0.11	0.59^**^	1	0.35^**^	−0.38	0.02
7.Narrative consistency	0.30^**^	0.29^**^	0.50^**^	0.34^**^	0.38^**^	0.35^**^	1	−0.41^**^	−0.23^*^
8.Counterarguing	−0.38^**^	0.43^**^	−0.55^**^	−0.08	−0.67^**^	−0.38^**^	−0.41^**^	1	0.22^*^
9.Psychological reactance	−0.01	−0.12	−0.01	−0.15	−0.1	0.02	−0.23^*^	0.22^*^	1

* p < 0.01

** *p < 0.001*.

**Table 4:** Descriptive statistics for all measured variables (study 2).

**Variable name**	**Mean**	***SD***
CMQ	5.01	1.23
Narrative related CTs	4.91	1.36
Enjoyment	4.75	1.53
Attentional focus	5.81	1.20
Overall plausibility	4.21	1.49
Plausibility US gov. assassinations	5.64	1.44
Plausibility US gov. concealing alien contact	4.33	1.88
Plausibility elite organizations conspiracy	4.09	1.80
Overall factuality	3.31	1.48
Factuality US gov. assassinations	4.63	1.58
Factuality US gov. concealing alien contact^*^	3.3	1.8
Factuality elite organizations conspiracy	3.3	1.7
Narrative consistency	5.06	1.07
Counterarguing	3.67	1.52
Psychological reactance	1.93	1.14

* *Median = 3, with skewness of.27, indicating positive asymmetry*.

**Table 5:** *F* and *p*-value for conspiracy beliefs related to the narrative, compared by conditions.

**Dependent variable**	***F*_(1, 164)_**	***p*-value**
“To protect some secrets, Western governments are willing to commit criminal acts, including assassinations”	0.12	0.27
“Above governments, there are organization that secretly organize worldwide chaos (international conflicts, financial crises, …) for their own profit”	0.20	0.66
“Some group are in possession of highly advanced military technology whose existence is kept secret”	0.88	0.35
Mean score of three beliefs	0.07	0.79

**Figure 2:**
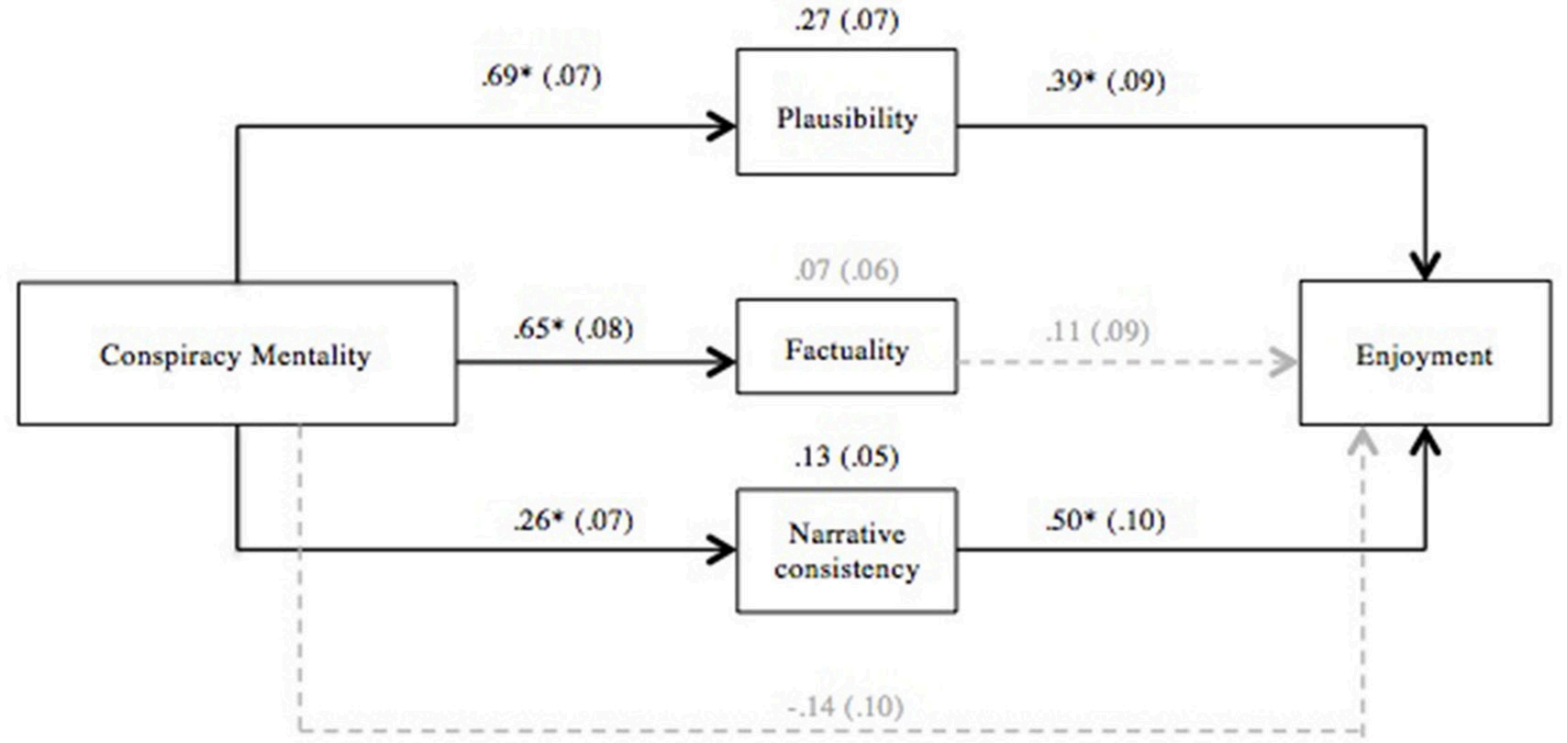
Mediation diagram with standardized regression coefficients, direct and indirect effects of conspiracy mentality on enjoyment, with bootstrap standard errors between parentheses. ^*^p < 0.001. Non-significant paths are marked in gray.

Principal Component Analysis with Varimax rotation confirmed the three-dimension factorial structure of the perceived realism items (see Appendix 2 for PCA statistics). In accordance with the exploratory analyses from Study 1, conspiracy mentality significantly predicted enjoyment of the video, *β* = 0.27, *t*(164) = 3.53, *p* = 0.001. Conspiracy mentality was strongly correlated to perceived plausibility, *r* = 0.57, *p* < 0.001, perceived factuality, *r* = 0.55, *p* < 0.001, and, slightly less, but still significantly, to narrative consistency, *r* = 0.30, *p* < 0.001. As for enjoyment of the narrative, it was strongly correlated with perceived plausibility, *r* = 0.51, *p* < 0.001, factuality, *r* = 0.39, *p* < 0.001, and narrative consistency, *r* = 0.50, *p* < 0.001, suggesting the hypothesized mediation. Multiple mediation analysis was performed using PROCESS custom dialog (Hayes, 2012), with confidence intervals of 95%, and 5,000 bootstrap samples. Enjoyment was encoded as dependent variable, and conspiracy mentality as independent variable. All dimensions of perceived realism were included as potential mediators. Significant indirect effects of conspiracy mentality were found for plausibility, IE = 0.27, CI [0.13, 0.40], and narrative consistency, IE = 0.13, CI [0.05, 0.23], but not for factuality, IE = 0.07, CI [−0.05, 0.20]. No direct effect of conspiracy mentality was detected, *t* = −1.39, *p* = 0.17, which suggests that dimensions of perceived realism fully mediated the effect of conspiracy mentality on enjoyment (see Figure 2).

#### Exploratory analyses

Counterarguing was negatively associated with enjoyment, *r* = −0.55, *p* < 0.001, conspiracy mentality, *r* = −0.38, *p* < 0.001, perceived plausibility, *r* = −0.67, *p* < 0.001, perceived factuality, *r* = −0.38, *p* < 0.001, narrative consistency, *r* = −0.41, *p* < 0.001. Hence, another indirect contribution of conspiracy mentality to enjoyment of the material may reside in the fact that subjects scoring high in conspiracy mentality engaged in less counteraguing while watching the video.

Given this result, we retested our first hypothesis, while controlling for counterarguing. We ran a hierarchical regression on endorsement of narrative-related conspiracy beliefs, introducing as independent variable counterarguing at step one, and condition at step two. Even in this configuration, there was no effect of narrative watching on the endorsement of narrative-related conspiracy beliefs, *t* = 0.11, *p* = 0.91.

## Bayesian meta-analysis

To examine whether the null hypothesis can be rejected, we adopted a Bayesian approach in order to determine whether we can confidently discard the possibility that watching the exposure material influences conspiracy beliefs (see Dienes, 2011).

In order to conduct a Bayesian meta-analysis of the two studies, we used the Jeffrey-Zellner-Siow specification (in line with Rouder et al., 2009), whereby the distribution of effect sizes is centered on zero, suggesting that small effect sizes are more frequent than larger ones (which makes them also more easily detectable). We used the BayesFactor package in R (Morey et al., 2015) to implement the approach. More specifically, we used this method on the mean of the three conspiracy beliefs related to the narrative across the two studies by relying on a meta-analytic *t*-test (Rouder and Morey, 2011). When doing so, we find a BF of 0.22, indicating that the null is 4.54 more likely given the data than the alternative hypothesis (i.e., presence of an effect of exposure to the narrative), which according to Rouder et al. (2009) constitutes “some evidence” for the null over the existence of an effect^3^.

In a second step, we considered a second alternative model in which the effect size is distributed only on the positive side. This corresponds to the narrative persuasion hypothesis that watching the episode has a positive effect on conspiracy beliefs. This model is associated with a BF of 0.05. Thus, the absence of an effect (the null) is 20 times more likely than the presence of a positive effect, which is conventionally considered as “strong evidence” in favor of the null over the alternative (Rouder et al., 2009).

Altogether, these Bayesian analyses allow us to discard with confidence the possibility that there is a positive effect of watching the narrative material and, more tentatively, that there is a boomerang effect.

## General discussion

Our attempt to experimentally explore the impact of conspiracist fiction on real-world conspiracy beliefs returned some unexpected results. Our primary hypotheses have been refuted. In two different samples (UK and Belgian), we find conclusive evidence that exposure to a strongly conspiracist narrative (i.e., an X-File episode) *does not* lead to greater endorsement of conspiracy beliefs consistent with the narrative. This finding goes both against a number of past narrative persuasion studies, as well as previous studies showing that exposure to conspiracy beliefs increases their endorsement (Douglas and Sutton, 2008; Jolley and Douglas, 2014; van der Linden, 2015). In the following sections, we explore reasons that may explain why this narrative, despite being enjoyed, failed to influence participants' beliefs. First, we propose explanations based on theoretical models of narrative persuasion (Green and Brock, 2000; Slater and Rouner, 2002; Busselle and Bilandzic, 2008), and comparisons with studies in which a narrative substantially impacted participants' beliefs on controversial topics (Butler et al., 1995; Slater et al., 2006; Iguarta and Barrios, 2012). Second, we examine how some characteristics of CTs may explain the absence of persuasive effect of the narrative material.

### Engagement in the narrative

The main theoretical models of narrative persuasion consider that narrative persuasion occurs via emotional and cognitive involvement in the narrative world (Moyer-Gusé and Dale, 2017). Several variables have been shown to influence the narrative involvement process, e.g., recipients' prior knowledge about the themes developed in the narrative (Green and Brock, 2000), perceived realism (Green, 2004; Busselle and Bilandzic, 2008), individuals' transportability (Mazzocco et al., 2010), whether or not the narrative fosters a strong imagery (Van Laer et al., 2013). Given the results of our experiments, we will now consider the generic question of perceived realism, and specifically, how this aspect of our narrative material may partly explain the present results.

#### Recipient's prior acquaintance with and perceived typicality of the narrative

According to Green (2004), being familiar with the themes dealt with in a narrative spurs transportation. For example, in a study using a narrative about a young gay man facing homophobic behaviors at a college fraternity reunion (Green, 2004), transportation, and therefore story-consistent attitude change, was found to be stronger when participants personally knew a gay person, and/or were personally familiar with the college fraternity system. Relatedly, perceived typicality, i.e., a dimension of perceived realism that designates the extent to which recipients think that the events depicted in the narrative fall within the range of the audience's real-life experience (Hall, 2003), has been shown to be positively associated with the strength of character identification (Cho et al., 2012).

Most previous studies that tested the impact of narratives on controversial attitudes (Slater et al., 2006; Iguarta and Barrios, 2012) used as exposure material audiovisual narratives that may have been directly or indirectly grounded in participants' personal experience. For example, the movie used in Iguarta and Barrios (2012) depicts the struggle of a little girl against cancer, whose treatment is impeded by the religious principles of her family. Conversely, our exposure material portrays events that can be hardly considered to fall within the audience's potential life experiences (hopefully). Such a distance between the narrative and the audience's personal experience may have led to less transportation, and less character identification. This hypothesis is also congruent with the narrative engagement approach (Busselle and Bilandzic, 2008), according to which narrative engagement can be diminished if the narrative is perceived not to reflect potential real-life situations.

#### Perceived factuality and plausibility of the narrative: a comparison with the impact of JFK

Even though perceived typicality favors character identification and therefore may facilitate narrative persuasion, there are examples of hardly typical narratives that were found to impact individuals' beliefs and attitudes. In a study anterior to the formulation of narrative persuasion theoretical models, the movie *JFK* (Stone, 1991) was shown to impact viewers' beliefs about CTs surrounding the assassination of John Fitzgerald Kennedy (Butler et al., 1995). Nevertheless, *JFK* did not include any supernatural aspects, and was moreover presented by its director as being based on rigorous journalistic inquiry. Such configuration may have strengthened the perception of plausibility and factuality of the narrative, and therefore facilitate movie consistent attitude changes. By contrast, even though a few elements in the X-Files episode were based on real events (e.g., references to the Patriot Act), it overwhelmingly consists of blatantly fictional material that was little plausible and obviously designed for entertainment purposes. The fact that both perceived factuality and plausibility were positively related to the endorsement of the narrative-related CTs partly supports an interpretation along these lines.

### The features of the persuasive message

Narrative persuasion studies predominantly aim at understanding the processing of entertainment-education fiction (e.g., Moyer-Gusé, 2008) and/or advertisings (Van Laer et al., 2013). As a result, models of narrative persuasion have devoted little attention to the characteristics of the persuasive content, and how these characteristics are likely to affect the persuasion process. In this section, we propose that two features of the persuasive content contained in the narrative may have prevented narrative persuasion to occur: the derogation of CTs, and the lack of relevant arguments supporting CTs in the narrative.

#### Derogation of conspiracy theories

CTs are widely derogated (e.g., Uscinski and Parent, 2014; Klein et al., 2015; Lantian et al., 2018). Evidence for the idea that CTs are derogated can also be found in the fact that people who endorse them disagree with the use of the expression “conspiracy theories” (Harambam and Aupers, 2016; Franks et al., 2017). The generalized derogation may, thus, set the topic of our narrative apart from the various attitudes, beliefs and behaviors promoted by narratives used in previous persuasion studies. In the few studies exposing participants to narratives with controversial topics (e.g., death penalty or gay marriage in Slater et al., 2006; or negative attitudes toward religion in Iguarta and Barrios, 2012) the topics were politically polarized, but not unanimously disregarded. Thus, the general derogation of conspiracy beliefs, the main element in our narrative material, may have impeded narrative persuasion. Besides, the narrative form in which CTs were presented in our study may explain why, contrary to studies using non-narrative exposure material (e.g., Douglas and Sutton, 2008; Jolley and Douglas, 2014; van der Linden, 2015), our conspiracy-related material did not increase endorsement of CTs. It is likely that the narrative form increased the perceived derogation of conspiracy beliefs.

#### Lack of relevant arguments supporting the conspiracy beliefs

According to the E-ELM (Slater and Rouner, 2002), a persuasive message put in the form of a narrative reduces targets' resistance to persuasion, therefore increasing its impact. Hence, it may not be mere transportation in a narrative world that makes people develop story-consistent attitudes and beliefs (e.g., Green and Brock, 2000; Van Laer et al., 2013), but rather the fact that being entertained and engaged in a narrative increases the recipients' receptivity to a persuasive message embedded in the narrative. According to the TIM, the strength of arguments plays a minor role in the narrative persuasion process, for the processing of arguments requires working memory resources that people enjoying a narrative momentarily lack (Green and Brock, 2002). However, it has been recently suggested that narratives that included strong arguments about a topic were more persuasive than narratives that included weak arguments, particularly when subjects were initially skeptical about the point advocated in the stories (Schreiner et al., 2018). Since CTs are controversial in nature, the last point may be particularly important to understand our results: when a persuasive message is likely to generate skepticism in the narrative recipients, embedding strong arguments in the narrative may be a necessary precondition for persuasion to occur.

In our exposure material, the characters expose a theory that is supported by pieces of evidence that the government has been secretly using alien technology against the population. Since participants considered the “alien” element of the plot to be relatively poorly based on facts (see Table 4), they may have considered that the advocated CT did not reflect actual events. By contrast, while the movie Camino does not explicitly enunciate arguments supporting the belief that “Opus Dei is a harmful religious organization” (Iguarta and Barrios, 2012), its story may have served as an implicit argumentative support by portraying the potential dramatic implications of the Opus Dei's lifestyle principle. Such argumentative support may be necessary for attitude change to occur.

The question of the presence of explicit and implicit arguments brings up the matter of the distinction between narrative and non-narrative persuasion. While traditional approaches to persuasion give importance to the processing of arguments in the attitude change process (e.g., Petty and Cacioppo, 1986), models of narrative persuasion devote much less attention to this aspect, for they tend to consider that transportation, or narrative engagement, is the primary vehicle of persuasion when it comes to stories. Our study, like Schreiner et al. (2018), highlights the interest of developing an approach of narrative persuasion that takes into account the arguments that explicitly or implicitly support the beliefs and attitudes that are depicted in the narrative.

Such an approach may be particularly relevant to address the issue of narrative persuasion in the domain of CTs, since CTs rely on argumentation and causal reasoning (Keeley, 1999). Besides, being exposed to the peremptory statement of a CT (e.g., “Above governments, there are organizations that secretly organize worldwide chaos”) may exert a lesser influence on one's beliefs than being exposed to arguments supporting this theory, throughout a narrative. This assumption is corroborated by the fact that most studies examining the impact of CTs on individuals' have used as exposure material information supporting CTs, rather than mere statements of CTs (e.g., Douglas and Sutton, 2008; Jolley and Douglas, 2014; van der Linden, 2015).

### Conspiracy mentality, enjoyment, and perceived realism

Besides the absence of a persuasive impact of the narrative, both studies evidenced relations between conspiracy mentality and variables related to the narrative experience (enjoyment, cognitive elaboration, character being identified with, dimensions of perceived realism). These data suggest that, even though no narrative persuasion effect was observed, there is a relation between real-world conspiracy views and receptivity to conspiracist fiction. This relation was fully mediated by the fact that participants with higher scores of conspiracy mentality tended to find the narrative more realistic. This brings up several questions for future research.

Mainly, these results suggest that stories tend to be considered as more realistic when there is already a convergence between the recipients' ideology and the worldview conveyed in the narrative (e.g., beliefs held by the characters). Hence, while narrative persuasion models focus on the impact of narratives on individuals' beliefs and attitudes, the relation between participants' belief system and their processing of narratives appears to be bidirectional. While the E-ELM acknowledges that similarity between recipients of the narrative and characters facilitates identification and therefore fosters vicarious social learning (Bandura, 1986; Slater and Rouner, 2002), the question of the role of recipients' prior belief system (e.g., political attitudes) on the processing of narratives has been scarcely investigated in narrative persuasion research. Considering that a part of narrative persuasion research aims at examining the impact of narratives on political attitudes (Slater et al., 2006; Iguarta, 2010; Iguarta and Barrios, 2012), data showing such a relation between individuals' prior political attitudes and reception of politically connoted narratives deserve to draw attention.

### Limitations and perspectives

Our studies have limitations. First, since the experiments were performed online in an uncontrolled environment, various uncontrolled variables may have affected our results. Nonetheless, it could be argued that this element adds in ecological validity, as watching an episode of a TV-show online is a very common pastime activity.

Another limitation is that the exposure material used in the studies is very stereotypically conspiracist. Such a choice is justified given that the studies are the first to unify the topics of narrative persuasion and CTs, but it raises questions as for the generalizability of our results. Moreover, the arguments underpinning the belief system of the characters in our exposure material may have lacked the power to generate the expected attitude change in the recipients' beliefs. Hence, further research is needed to know to what extent our results can be generalized to other narratives dealing with the thematic of CTs. Specifically, examining how such narratives develop implicit and explicit arguments supporting their conspiratorial content is a research topic that would be worth investigating further. Such an approach would constitute a substantial contribution to both narrative persuasion research, which has up to recently neglected the potential role of argumentation in the narrative persuasion process (Schreiner et al., 2018), and research on CTs, which have scarcely investigated the question of arguments supporting CTs.

A last limitation is that, as explained in the introduction, a key feature of CTs is that they are underpinned by a general belief system. Narrative persuasion studies assess the impact of narratives on specific beliefs, rather than general attitudes that are relatively stable. Developing longitudinal approaches to narrative persuasion by developing experimental protocols that go beyond the mere exposure to a specific persuasive content could greatly strengthen our knowledge in this field. For the same reason, an open question remains whether indirect measures might be more adequate for assessing endorsement of CTs and related narrative persuasion effects, since a direct assessment, like the one we employed may be too transparent.

## Conclusion

In today's world, watching TV-shows and movies is probably among the most common pastime activities, alongside with playing video games and, to a lesser extent, reading novels. As a consequence, fiction is probably more present in our lives than it ever was. Hence, exploring the impact of fiction on the major social phenomenon of CTs is anything but trivial.

The present studies suggest that exposure to strongly conspiracist fiction does not lead to greater endorsement of CTs related to the narrative, contrary to the results of other research showing that narratives could influence endorsement of controversial beliefs and attitudes. Besides, they suggest that people who already espouse conspiracist worldviews may enjoy these narratives more, in part because they find them more plausible. Thus, while such fiction does not in and by itself change people's views on CTs, it may serve the function of supplying conspiracy believers with material that supports their worldview and, in the long run, buttresses their monological belief system. In this regard, longitudinal studies examining the impact of exposure to conspiracist series over much longer periods of time would be welcome. Finally, the relation between conspiracy mentality and the reception of a conspiracist narrative highlights the importance of paying more attention to how individuals' prior attitudes can influence the processing of a narrative.

## Ethics statements

The first study of the present research was conducted in the context of a master thesis at the Department of psychology and educational sciences of the Université Libre de Bruxelles, Belgium. Following the institution's rules regarding master theses in psychology, a form called “formulaire d'information éthique,” designed by the Faculty Ethics Committee (Comitê d'Ethique Facultaire, affiliated with the Faculté des Sciences Psychologiques et de l'Education) was submitted to the department. This form (accessible at the following URL: http://www.ulb.be/facs/psycho/ethique.html) was filled and signed by both the master student (first author of this paper) and his supervisor (Pr. Olivier Klein). It included questions allowing to gauge possible ethical issues associated with the study. In the context of master theses, the Committee does not conduct a formal approval procedure unless this is explicitly requested. Given the nature of the study, which does not involve deception or harm to the participants, it was deemed unnecessary to solicit formal ethical approval. The aforementioned procedure complies with belgian research regulation rules. Given the fact that the experiment was conducted online, participants could interrupt their participation at any moment. Therefore, even though participants did not give any written nor oral formal informed consent before engaging in the experiments, consent was given by virtue of survey completion.

## Author contributions

Most of the paper was written by KN. He reviewed the literature, designed the studies, recruited participants, performed the statistical analyses. MP provided substantial contributions to the discussion and conclusion segments, corrected the draft, intermediate and final versions of this manuscript. She also provided many helpful corrections and suggestions. OK supervised the design of the studies, provided valuable help with statistical analyses and their interpretation, and performed the Bayesian meta-analysis. He also implemented a number of corrections, made helpful suggestions, and approved the intermediate and final versions of this manuscript.

### Conflict of interest statement

The authors declare that the research was conducted in the absence of any commercial or financial relationships that could be construed as a potential conflict of interest.

## Footnotes

^1^To name a few: 3 Days of the Condor (Pollack, 1975), Enemy of the State (Scott, 1998), This Perfect Day (Levine, 1970), The Da Vinci Code (Brown, 2003), The Matrix (Wachowski and Wachowski, 1999), X-Files (Carter, 1993), Stranger Things (Duffer and Duffer, 2016), House of Cards (Willimon, 2013), Utopia (Kelly, 2013-2014), Lost (Lieber et al., 2004-2010), Bioshock Infinite (Irrational Games, 2013), Metal Gear Solid (Kojima, 1998).

^2^The experiment was conducted in early 2016. After completing data collection, we found out that Lantian et al. (2016) had developed and validated a French version of the conspiracy mentality scale. Even though we did not use their translation in our experiment, we deemed necessary to mention its existence.

^3^The scale is the following: odds >3 (or < 0.33) can be consider as “some evidence,” odds >10 (or < 0.10) as “strong evidence,” and odds >30 (or < 0.033) as “very strong evidence” for one hypothesis over another.

## Appendix 1

**Table:** Principal Component Analysis (Varimax rotation) of the identification with a character scale.

	**Factor**		
**Items**	**1**	**2**	**3**
- Je me suis senti.e semblable au personnage, proche de lui.	0.78		
- Je me suis dit que j'aimerais être, ou agir comme le personnage.	0.84		
- Je me suis identifié.e au personnage.	0.75		
- Je me suis senti.e ≪ devenir le personnage ≫.	0.63		
- J'avais l'impression de vivre l'histoire vécue par le personnage.	0.77		
- J'ai compris la manière dont le personnage agissait, pensait, ressentait.		0.82	
- J'ai essayé de voir les choses du point de vue du personnage.		0.88	
- J'ai essayé d'imaginer les sentiments, les émotions du personnage.		0.83	
- J'ai ressenti les réactions émotionnelles du personnage.			0.60
- Je me souciais de ce qui allait arriver au personnage.			0.75
- Je me suis senti.e touché.e émotionnellement par ce que ressentait le personnage.			0.88
- Je me suis demandé ce que je ferais à la place du personnage.			0.5
- Je me souciais de ce qui allait arriver au personnage.			0.75
Eigenvalue	6.38	1.65	1.33
% explained variance	49.06	12.65	10.22

## Appendix 2

**Table:** Principal Component Analysis (Varimax rotation) of the perceived realism scales.

	**Factor**		
**Items**	**1**	**2**	**3**
Overall, the video shows things that could happen in real life.	0.64	
Overall, events portrayed in the video are potential real life situation.	0.66	
Real people would not do any of the things shown in the video (R).	0.87	
Never in real life would anything showed in the video happen (R).	0.86	
Many elements in the video are based on facts.		0.84
The video shows many things that really happened.		0.83
There are things that are shown in the video that actually happen in the real world.		0.71
The video showed a coherent story.			0.80
Parts of the video were contradicting each other (R).			0.53
The story portrayed in the video made sense.			0.86
The events in the video had a logical flow.			0.84
Eigenvalue	5.12	1.81	1.34
*% explained variance*	46.58	16.45	12.16

## Abbreviations

CT: Conspiracy Theory
E-ELM: Extended-Elaboration Likelihood Model (Slater and Rouner, 2002)
TIM: Transportation Imagery Model (Green and Brock, 2000).
